# Plasma Free Amino Acid Responses to Intraduodenal Whey Protein, and Relationships with Insulin, Glucagon-Like Peptide-1 and Energy Intake in Lean Healthy Men

**DOI:** 10.3390/nu8010004

**Published:** 2016-01-04

**Authors:** Natalie D. Luscombe-Marsh, Amy T. Hutchison, Stijn Soenen, Robert E. Steinert, Peter M. Clifton, Michael Horowitz, Christine Feinle-Bisset

**Affiliations:** 1 NHMRC Centre of Research Excellence in Translating Nutritional Science to Good Health, The University of Adelaide Discipline of Medicine, Adelaide 5000, Australia; amy.hutchison@adelaide.edu.au (A.T.H.); stijn.soenen@adelaide.edu.au (S.S.); robert.steinert@adelaide.edu.au (R.E.S.); Peter.Clifton@unisa.edu.au (P.M.C.); michael.horowitz@adelaide.edu.au (M.H.); christine.feinle@adelaide.edu (C.F.-B.); 2 CSIRO Food and Nutrition, PO Box 10041 Adelaide BC, Adelaide SA 5000, Australia; 3 School of Pharmacology and Medical Sciences, University of South Australia, Adelaide 5001, Australia

**Keywords:** dairy, whey protein hydrolysate, insulinotropic response, glycemic control, appetite regulation, human

## Abstract

This study determined the effects of increasing loads of intraduodenal (ID) dairy protein on plasma amino acid (AA) concentrations, and their relationships with serum insulin, plasma glucagon-like peptide-1 (GLP-1) and energy intake. Sixteen healthy men had concentrations of AAs, GLP-1 and insulin measured in response to 60-min ID infusions of hydrolysed whey protein administered, in double-blinded and randomised order, at 2.1 (P2.1), 6.3 (P6.3) or 12.5 (P12.5) kJ/min (encompassing the range of nutrient emptying from the stomach), or saline control (C). Energy intake was quantified immediately afterwards. Compared with C, the concentrations of 19/20 AAs, the exception being cysteine, were increased, and this was dependent on the protein load. The relationship between AA concentrations in the infusions and the area under the curve from 0 to 60 min (AUC_0–60 min_) of each AA profile was strong for essential AAs (*R*^2^ range, 0.61–0.67), but more variable for non-essential (0.02–0.54) and conditional (0.006–0.64) AAs. The AUC_0–60 min_ for each AA was correlated directly with the AUC_0–60 min_ of insulin (*R*^2^ range 0.3–0.6), GLP-1 (0.2–0.6) and energy intake (0.09–0.3) (*p* < 0.05, for all), with the strongest correlations being for branched-chain AAs, lysine, methionine and tyrosine. These findings indicate that ID whey protein infused at loads encompassing the normal range of gastric emptying increases plasma concentrations of 19/20 AAs in a load-dependent manner, and provide novel information on the close relationships between the essential AAs, leucine, valine, isoleucine, lysine, methionine, and the conditionally-essential AA, tyrosine, with energy intake, insulin and GLP-1.

## 1. Introduction

Calorie-controlled diets with moderately increased protein content are effective in the management of obesity due to their capacity to (i) suppress appetite [1] and, under *ad-libitum* feeding conditions, reduce energy intake [2]; (ii) promote loss of fat while preserving muscle mass [3,4]; (iii) increase insulin release and reduce postprandial glycemic excursions [1,3]; and (iv) improve lipid metabolism and blood pressure [1,3]. These benefits, however, are often not sustained for more than 12 months, and hence, greater understanding of the mechanisms underlying these benefits is required.

Oral and intraduodenal (ID) administration of dietary protein, including whey protein isolate (in both un-hydrolysed and hydrolysed form), like lipid and carbohydrate, modulates gastrointestinal (GI) motor activity and hormone release in a nutrient load-dependent manner [5,6,7]. In turn, these GI factors influence gastric emptying [8], postprandial glycaemia [9] and energy intake [5,6,7]. While whey protein isolate, co-ingested with carbohydrate, improves glycaemia by slowing gastric emptying and stimulating the release of glucagon-like peptide-1 (GLP-1) and/or glucose-dependent insulinotropic polypeptide (GIP) as well as insulin [10], our recent observations indicate that the effects of lipid–protein combinations on these gut hormones are related directly to the amount of lipid, and the effects on insulin and glucagon are related to the amount of protein, whereas the effects on energy intake appear dependent on a threshold load of ~12.5 kJ/min (or 3 kcal/min) of either nutrient being delivered to the duodenum [11]. This study also demonstrated that the effects of ID infused hydrolysed whey protein isolate (which did not contain fat or carbohydrate and was delivered at 12.5 kJ/min) on the GI factors were weaker than those of isocaloric lipid [11] suggesting the suppression of energy intake by protein may be dependent on additional mechanisms.

Several studies have reported modest associations between increased postprandial plasma essential amino acid (AA) concentrations, particularly the branched chain AAs, leucine, isoleucine and valine, with either an increased release of some gut hormones (particularly GLP-1 and GIP), insulin, or reduced energy intake [12,13,14]. Moreover, there is some evidence that other essential AAs, including lysine, threonine, phenylalanine, tryptophan, histidine, the conditionally-essential AAs, glutamine, arginine and tyrosine, and the non-essential AA, glutamate, may activate peripheral and central mechanisms involved in the regulation of energy intake and glucose homeostasis, although information is limited [15].

The aims of this study were to determine: (1) the effects of increasing loads of hydrolysed whey protein administered ID (thus mimicking the entry of the nutrient into the duodenum and bypassing any orosensory influences and inter-individual variations in gastric emptying), at rates reflective of the normal range of gastric emptying, on plasma concentrations of free AAs that were present within the whey; and (2) the relationships between plasma AA concentrations with insulin and GLP-1 concentrations and energy intake, outcomes that we have previously reported [7]. We hypothesised that hydrolysed whey protein infused ID at loads lower than (2.1 kJ/min (P2.1)), similar to (6.3 kJ/min (P6.3)), and toward the upper end (12.5 kJ/min (P12.5)), of normal gastric emptying rates would modulate the release of free AAs into the blood in a load-related manner, and leucine, isoleucine, valine, and potentially other AAs, would be related to the insulin and GLP-1 responses and energy intake.

## 2. Subjects and Methods

### 2.1. Subjects

Sixteen healthy lean men (mean age 27 ± 3 years, range 19–54 years; BMI 22.1 ± 0.6 kg/m^2^; range 18.5–24.8 kg/m^2^) participated in the study, as described [7]. In addition to the original power calculation [7], we calculated that *n* = 16 would allow us to detect relationships of *r* ≥ 0.5 between increasing loads of protein with total plasma AAs, and increasing plasma total AA concentrations with energy intake, plasma insulin and GLP-1 [5,6]. The Royal Adelaide Hospital Research Ethics Committee approved the study protocol, and the study was registered as a clinical trial with the Australia and New Zealand Clinical Trial Registry (www.anzctr.org.au, registration number 12610000376044). All subjects provided written, informed consent, prior to their inclusion.

### 2.2. Study Outline

While the aim of the original study was to evaluate the effects of increasing loads of ID infusion of hydrolysed whey protein at loads of (i) 2.1 kJ/min “P2.1”; (ii) 6.3 kJ/min “P6.3”; or (iii) 12.5 kJ/min “P12.5”; or (iv) a saline control “C”, at a rate of 4 mL/min for 60 min, on antropyloroduodenal motility (APD), and gut hormone, insulin, glucose, appetite and energy intake responses [7], we have now evaluated the effects on plasma free AAs to determine their relationships with GLP-1, insulin, and energy intake [7].

### 2.3. Intraduodenal Infusions

The infusion solutions were prepared as described [7]. In brief, hydrolysed whey protein (Whey Protein Hydrolysate 821, Fonterra Co-Operative Group Ltd, Auckland, New Zealand) was dissolved in varying amounts of saline and water to achieve the desired loads (*i.e.*, 2.1, 6.3 and 12.5 kJ/min, which equated to 8, 24 and 48 g of protein over 60 min) and to ensure they were all a total volume of 240 mL and equi-osmolar (~640–676 mOsmol/L). As a component of dairy products, whey protein is common in the diet and has been shown to be the most satiating protein source when consumed orally [16,17]. We have used whey protein in our previous studies evaluating the role of macronutrients on GI function and appetite [7,10,11]. Hydrolysed whey was selected because it contains di- and tri-peptide fractions as well as larger peptides and some amino acids, and hence, more closely resembles partially digested protein, that would be entering the small intestine following oral protein ingestion [18,19]. Infusions were prepared on the morning of each study by an investigator who was not involved in the data analysis. The infusion apparatus was covered at all times so that both the primary investigator and the subject were blinded to the treatment. Table 1 depicts the specific AA composition of the hydrolysed whey protein.

**Table 1:** The amino acid (AA) composition of the whey protein infusion solutions ^a,b,c^.

AA	Treatment		
	P2.1	P6.3	P12.5
	***g (mol/L)***		
Glutamic acid	1.3 (36.3)	3.8 (108.9)	7.7 (217.8)
Leucine	0.9 (29.7)	2.8 (89.1)	5.6 (178.2)
Aspartic acid	0.8 (26.5)	2.5 (79.3)	5.1 (158.7)
Lysine	0.8 (23.4)	2.5 (70.3)	5.0 (140.5)
Alanine	0.4 (20.8)	1.3 (62.4)	2.7 (124.8)
Valine	0.4 (14.7)	1.2 (44.2)	2.5 (88.4)
Proline	0.4 (14.5)	1.2 (43.4)	2.4 (86.9)
Isoleucine	0.4 (14.1)	1.3 (42.4)	2.7 (84.8)
Threonine	0.4 (13.2)	1.1 (39.5)	2.3 (79.1)
Serine	0.3 (9.9)	0.8 (29.6)	1.5 (59.2)
Phenylalanine	0.3 (7.8)	0.9 (23.4)	1.9 (46.7)
Tyrosine	0.3 (7.3)	0.9 (21.9)	1.9 (43.7)
Arginine	0.3 (5.9)	0.8 (17.8)	1.5 (35.7)
Glycine	0.2 (10.5)	0.6 (31.4)	1.1 (62.8)
Cysteine	0.2 (6.5)	0.6 (19.5)	1.1 (38.9)
Methionine	0.2 (4.9)	0.5 (14.5)	1.0 (29.1)
Histidine	0.2 (4.7)	0.5 (14.0)	1.0 (28.0)
Tryptophan	0.2 (3.9)	0.6 (11.6)	1.1 (23.1)
Glutamine	Not reported		
Asparagine	Not reported		
*Total*	*8.0 (254)*	*24.0 (763)*	*48.0 (1526)*

^a^ The amino acid (AA) composition of “Hydrolysed Whey Protein 821” was provided by Fonterra Co-Operative Group Ltd, Auckland, New Zealand, and glutamine and asparagine were not reported in protein product specification; ^b^ The infusions (total volume 240 mL) consisted of 2.1 kJ/min (P2.1), 6.5 kJ/min (P6.3), or 12.5 kJ/min (P12.5) of whey protein, each infused at 4 mL/min for 60 min; ^c^ The AAs are presented in order of most to least abundant g present within the protein infusions and glutamic acid and aspartic acid include the contribution from asparagines and glutamine in this product specification.

Subjects were studied on four occasions, each separated by 3–10 days, in a randomized, double-blind, cross-over design. Randomisation was performed by an investigator not involved in the assessments using the free software “Research Randomiser”, and the investigator performing the assessments was blinded to the randomisation and preload conditions. Subjects were provided with a standardised evening meal to consume the night prior to each study day, and were instructed to abstain from all food and drinks and to refrain from strenuous physical activity, until attending the laboratory at the Discipline of Medicine at 8:30 a.m. Upon arrival, a small-diameter (OD: 3.5 mm) manometric catheter (total length: 100 cm, Dentsleeve International, Mui Scientific, Mississauga, ON, Canada) was inserted into the stomach through an anesthetised nostril and allowed to pass into the duodenum by peristalsis. The catheter contained 16 side-holes spaced at 1.5 cm intervals, with an additional channel used for ID infusions located 11.75 cm distal to the end of the sleeve sensor (14.5 cm from the pylorus), and was positioned as described previously [7]. Once the catheter was positioned correctly (which took between 15 and 45 min), fasting motility was monitored until the occurrence of a phase III of the interdigestive migrating motor complex. Immediately after cessation of phase III activity, an intravenous cannula was placed in a right forearm vein for blood sampling. At *t* = −15 min (during motor quiescence, *i.e.*, phase I of the migrating motor complex), a baseline blood sample (14 mL) was taken and a visual analogue scale questionnaire (VAS) to assess appetite perceptions and GI symptoms completed by the subject. Baseline measurement of APD motility also commenced. At *t* = 0 min, the 60-min ID infusion of one of the treatments commenced. During the infusion, APD motility was monitored continuously, and VAS ratings and blood samples were obtained at 15-min intervals. At *t* = 60 min, the infusion was terminated and the ID catheter removed. Subjects were then presented with a standardised cold, buffet-style meal, in excess of what they would be expected to consume, and instructed to eat freely for up to 30 min (*t* = 60–90 min) until comfortably full [20]. The composition of the buffet meal has been described [20].

### 2.4. Measurements

*Plasma free AA and GLP-1 and serum insulin concentrations:* 10-mL venous blood samples were collected in ice-chilled EDTA-treated tubes for plasma AA and GLP-1 analysis, and 4-mL samples in serum-Z tubes containing clotting beads (Sarstedt Australia Pty Ltd, Adelaide, Australia) for insulin. Plasma/serum was separated by centrifugation at 3200 rpm for 15 min at 4 °C, and stored at −70 °C for later analysis. Plasma free AAs concentrations (mmol/L) of asparagine, aspartic acid, alanine, arginine, cysteine, glutamine, glutamic acid, glycine, histidine, isoleucine, leucine, lysine, methionine, phenylalanine, proline, serine, threonine, tryptophan, tyrosine and valine, were analysed using precolumn derivatization with 6-aminoquinolyl-N hydroxysuccmimidyl carbamate (AQC). The derivatives were then separated and quantified by reversed-phase high-performance liquid chromatography (HPLC). The AAs (with the exception of tryptophan) were detected by fluorescence, whereas tryptophan required UV detection. Before derivatisation, 100 µL of plasma samples were diluted 1:1 with internal standard solution (Norvaline) and deproteinised by ultra-filtration through a membrane with 10 kDa nominal molecular weight cut-off (Ultrafree MC with PL-10 membrane, Millipore, MA, USA). AAs contained in the filtrate (100 µL) were labelled using the Waters AccQTag™ chemistry and analysed using a Waters Acquity™ UPLC system (Waters Corporation, MA, USA) [21]. Only single analysis of each AA was conducted. The analysis was performed at the Australian Proteome Analysis’s Facility established under the Australian Government’s National Collaborative Research Infrastructure Strategy (NCRIS). All analyses were performed by the same technician. Plasma GLP-1 (pmol/L) and serum insulin (mU/L) were measured, as described [7].

*Energy intake:* The amount eaten (g) was quantified by weighing the buffet meal before and after consumption, and energy (kJ) was then calculated using commercially available software (Foodworks, Xyris Software, version 3.01, Highgate Hill, Australia).

### 2.5. Data and Statistical Analyses

Statistical analyses were performed using SPSS software (version 19.0; SPSS Inc, IBM, New York, NY, USA). Baseline plasma AA concentrations were analysed using one-way ANOVA with protein load as the within-subject factor. The effect of protein load on the net incremental area under the curve (AUC_0–60 min_) for each AA (calculated using the trapezoidal rule) was analysed by one-way ANOVA with protein load as the fixed factor. *Post-hoc* comparisons, adjusted for multiple comparisons using Bonferroni’s correction, were performed when ANOVAs revealed significant effects. The effects of increasing loads of protein on insulin and GLP-1 concentrations and on energy intake at a subsequent meal have been reported [7], and additional analyses of the AUC for insulin and GLP-1 were done to determine relationships between these outcomes with the AUC for each of the 20 AAs. Relationships between the AUC for each AA and either protein load, total AA concentration within the infusion, energy intake or AUC for insulin and GLP-1, respectively, were evaluated using linear within-subject correlations *(r)* with AUC for each AA profile as the dependent, subject as a fixed factor and the other variable as a covariate. Relationships between each AA with energy intake and AUCs for insulin and GLP-1, respectively, were ranked in order of strongest to weakest, using R square values. Statistical significance was accepted at *p* < 0.05; all data are presented as mean ± SEM.

## 3. Results

### 3.1. Plasma AA Concentrations

Baseline concentrations of individual and total AAs did not differ between test days (Table 2).

**Table 2:** Plasma amino acid (AA) concentrations at baseline prior to commencing each infusion ^a,b,c^.

AA	C	Treatment			*p Value* ^c^
		P2.1	P6.3	P12.5	
	*µmol/L*				
Glutamine	520	530	540	540	>0.05
Alanine	240	240	240	270	>0.05
Valine	190	190	200	190	>0.05
Glycine	180	180	190	190	>0.05
Proline	150	150	150	160	>0.05
Lysine	150	150	150	150	>0.05
Threonine	200	100	100	100	>0.05
Leucine	100	100	100	100	>0.05
Serine	90	90	90	90	>0.05
Glutamic acid	80	90	90	90	>0.05
Arginine	80	80	80	80	>0.05
Histidine	60	70	70	70	>0.05
Isoleucine	50	50	50	50	>0.05
Tyrosine	50	50	50	50	>0.05
Asparagine	40	50	50	50	>0.05
Phenylalanine	40	40	40	40	>0.05
Methionine	020	20	20	20	>0.05
Aspartic acid	3	3	3	3	>0.05
Tryptophan	3	3	3	3	>0.05
Cysteine	2	2	2	2	>0.05
*Total*	*2130*	*2200*	*2200*	*2230*	*>0.05*

^a^ Data are means, and the amino acids (AAs) have been presented in order of most to least concentrated, *n* = 16; ^b^ The infusions consisted of either saline (C), or of 2.1 kJ/min (P2.1), 6.5 kJ/min (P6.3), or 12.5 kJ/min (P12.5) of hydrolysed whey protein, each infused at 4 mL/min for 60 min; ^c^ Main effect of protein load was determined by one-way ANOVA and *post-hoc* comparisons between two loads of protein were done using Bonferroni’s correction; statistical significance was accepted at *p* < 0.05.

**Figure 1:**
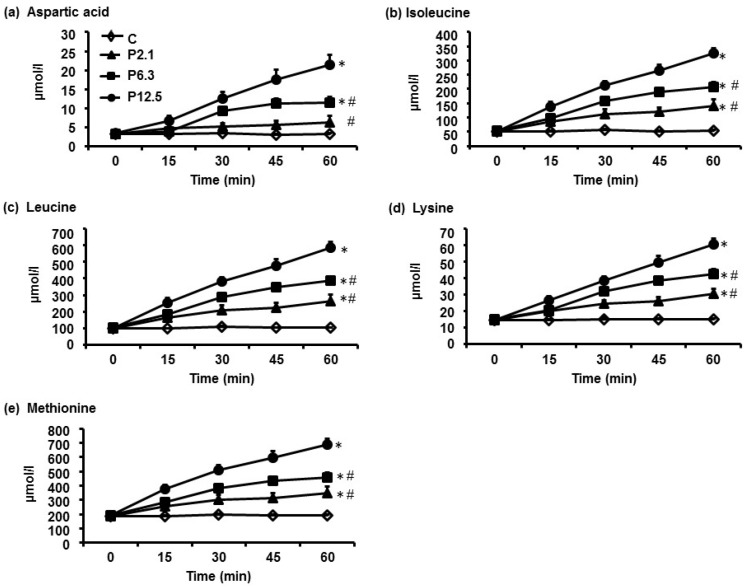
The temporal profiles of (**a**) aspartic acid; (**b**) isoleucine; (**c**) leucine; (**d**) lysine; and (**e**) methionine, the five amino acids whose plasma concentrations increased the most in response to increasing loads of ID protein. Data are means ± SEM, *n* = 16. The infusions consisted of either saline (C), or of 2.1 kJ/min (P2.1), 6.5 kJ/min (P6.3), or 12.5 kJ/min (P12.5) of hydrolysed whey protein, each infused at 4 mL/min for 60 min. Main effect of protein load was determined by one-way ANOVA and *post-hoc* comparisons between two loads were done using Bonferroni’s correction; statistical significance was accepted at *p* < 0.05. * Significantly different from C. # Significantly different from P12.5 (*p* < 0.05).

**Figure 2:**
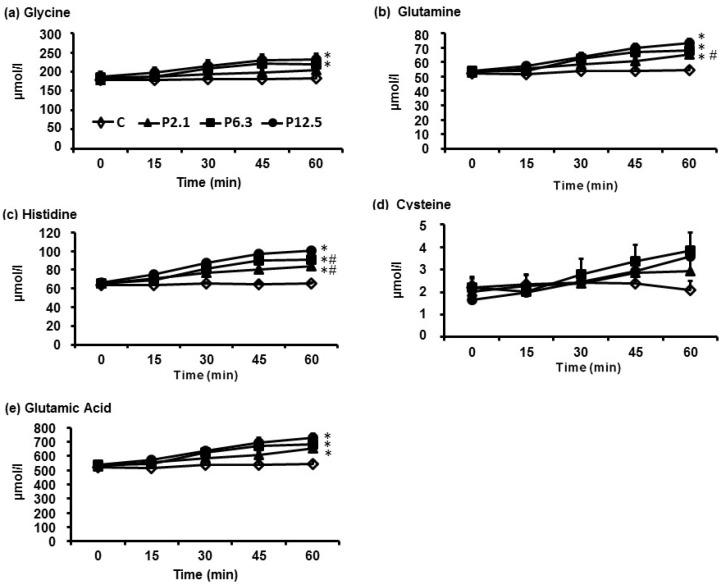
The temporal profiles of (**a**) glycine; (**b**) glutamine; (**c**) histidine; (**d**) cysteine; and (**e**) glutamic acid, the five amino acids whose plasma concentrations increased the least in response to increasing loads of ID protein. Data are means ± SEM, *n* = 16; The infusions consisted of either saline (C), or of 2.1 kJ/min (P2.1), 6.5 kJ/min (P6.3), or 12.5 kJ/min (P12.5) of hydrolysed whey protein, each infused at 4 mL/min for 60 min; Main effect of protein load was determined by one-way ANOVA and *post-hoc* comparisons between two loads were done using Bonferroni’s correction; statistical significance was accepted at *p* < 0.05. * Significantly different from C. # Significantly different from P12.5 (*p* < 0.05).

In response to increasing loads of ID whey protein, the greatest increases in plasma concentrations over the 60-min infusion were observed for aspartic acid, isoleucine, leucine, lysine and methionine (Figure 1), whereas the smallest increases were observed for glycine, glutamine, histidine, glutamic acid and cysteine (Figure 2). The AUC_0–60min_ of each of the 20 AA profiles in response to increasing protein loads are depicted in Table 3. There was a significant main effect of protein load on plasma concentrations of 19/20 AAs (*p* < 0.001), with the exception of cysteine. *Post hoc*-analyses revealed that the AUC_0–60min_ of plasma concentrations of 16/20 AAs (*p* < 0.05 for all), except tryptophan, alanine, glycine and cysteine, were greater during P2.1 compared with C. While the AUC_0–60min_ of the plasma concentrations of each of the 20 AAs did not differ between P2.1 and P6.3, the AUC_0–60min_ of all AA profiles, apart from glutamic acid, glycine and cysteine, were greater during P12.5 than during P2.1 (*p* < 0.05 for all). In addition, the AUC_0–60min_ of 11/20 AAs, the exceptions being aspartic acid, threonine, asparagine, alanine, serine, glutamic acid, glutamine, glycine and cysteine, were greater during P12.5 than during P6.3 (*p* < 0.05 for all).

**Table 3:** Plasma amino acid (AA) responses (AUC_0–60 min_) ordered from the strongest to weakest response following the infusions, relative to control ^a,b,c^.

AA	C	Treatment			*p Value ^c^*
		P2.1	P6.3	P12.5	
	***µmol.60 min/L***				
Aspartic acid	200	300 ^d,e^	500 ^d^	7000 ^d^	<0.001
Isoleucine	3200	6200 ^d,e^	8600 ^d,e^	12,100 ^d^	<0.001
Leucine	6100	11,700 ^d,e^	15,900 ^d,e^	21,900 ^d^	<0.001
Lysine	9000	14,000 ^d,e^	18,000 ^d,e^	22,800 ^d^	<0.001
Methionine	1100	1700 ^d,e^	2100 ^d,e^	2900 ^d^	<0.001
Tryptophan	2000	300 ^e^	300 ^d,e^	4000 ^d^	<0.001
Tyrosine	2800	3800 ^d,e^	4500 ^d,e^	5800 ^d^	<0.001
Valine	11,300	14,600 ^d,e^	17,500 ^d,e^	20,800 ^d^	<0.001
Phenylalanine	2500	3200 ^d,e^	3600 ^d,e^	4500 ^d^	<0.001
Threonine	5800	7600 ^d,e^	9100 ^d^	10,500 ^d^	<0.001
Asparagine	2700	3500 ^d,e^	4200 ^d^	4800 ^d^	<0.001
Proline	8800	10,700 ^d,e^	12,100 ^d,e^	14,800 ^d^	<0.001
Alanine	14,500	17,000 ^e^	19,200 ^d^	23,100 ^d^	<0.001
Arginine	4600	6100 ^d,e^	700 ^d,e^	8400 ^d^	<0.001
Serine	5200	6500 ^d,e^	7400 ^d^	8000 ^d^	<0.001
Glutamic acid	4900	6100 ^d^	6200 ^d^	7100 ^d^	<0.001
Histidine	3900	4500 ^d,e^	4800 ^d,e^	5100 ^d^	<0.001
Glutamine	31,900	35,100 ^d,e^	36,700 ^d^	38,100 ^d^	<0.001
Glycine	10,800	11,600	12,300 ^d^	12,800 ^d^	<0.001
Cysteine	100	100	200	100	>0.05
*Total*	*129,000*	*165,000* ^d,e^	*190,000* ^d,e^	*225,000* ^d^	*<0.001*

^a^ Data are means, and the amino acids (AAs) have been presented in order of most to least concentrated, *n* = 16; ^b^ The infusions consisted of either saline (C), or of 2.1 kJ/min (P2.1), 6.5 kJ/min (P6.3), or 12.5 kJ/min (P12.5) of hydrolysed whey protein, each infused at 4 mL/min for 60 min; ^c^ Main effect of protein load was determined by one-way ANOVA, and *post-hoc* comparisons between two loads were done using Bonferroni’s correction; statistical significance was accepted at *p* < 0.05; ^d^ Significantly different from C (*p* < 0.05); ^e^ Significantly different from P12.5 (*p* < 0.05).

Strong positive relationships between protein load and the resultant AUC_0–60min_ of the plasma concentrations of all essential AAs were evident (*i.e.*, range of *R*^2^ values was 0.58–0.69; *p* < 0.001 for all), whereas the strength of the relationships was more variable for non-essential (*i.e.*, 0.28–0.55; *p* < 0.05 for all) and conditional (*i.e.*, 0.01–0.66; *p* < 0.05 for all) AAs. In addition, plasma concentrations of essential AAs reflected closely their concentrations in the infused whey protein (*i.e.*, range of *R*^2^ values was 0.61–0.67; *p* < 0.001 for all), whereas the relationships were more variable for non-essential (*i.e.*, 0.02–0.54; *p* < 0.001 for all) and conditional (*i.e.*, 0.006–0.64; *p* < 0.01 for all; the exception being cysteine which was not related) AAs.

### 3.2. Serum Insulin and Plasma GLP-1 Concentrations, and Energy Intake and Amount of Food Consumed at the Buffet Meal

For the purpose of the current analysis, the temporal profiles of insulin and GLP-1 that were reported previously [7] have been expressed as AUCs. Table 4 depicts the AUCs_0–60min_ of the serum insulin, and plasma GLP-1, concentrations, and energy intake and the amount of food consumed at the buffet meal, in response to increasing loads of protein.

**Table 4:** Serum insulin and plasma GLP-1 responses (AUCs_0–60 min_), and energy intake and amount of food consumed at the buffet meal, in response to increasing loads of ID protein ^a,b,c^.

Hormone and Food Intake Response	C	P2.1	P6.3	P12.5	*p* Value ^c^
Insulin (mU.60min/L)	150 ± 19	354 ± 94 ^d^	721 ± 99 ^d^	1087 ± 145 ^d,e^	<0.05
GLP-1 ( mmol.60min/L)	1489±113	1698 ± 142	2053 ± 159 ^d,f^	2147 ± 171 ^d,f^	<0.05
Energy intake (buffet; kJ)	5173 ± 464	4981 ± 473	4504 ± 523 ^g^	3814 ± 502 ^d,e,f^	<0.05
Amount of food eaten (g)	1261 ± 102	1196 ± 104	1109 ± 118	1009 ± 107	0.08

^a^ Data are means, and the amino acids (AAs) have been presented in order of most to least concentrated, *n* = 16; ^b^ The infusions consisted of either saline (C), or of 2.1 kJ/min (P2.1), 6.3 kJ/min (P6.3), or 12.5 kJ/min (P12.5) of hydrolysed whey protein, each infused at 4 mL/min for 60 min; ^c^ Main effect of protein load was determined by one-way ANOVA and *post-hoc* comparisons between two loads were done using Bonferroni’s correction; statistical significance was accepted at *p* < 0.05; ^d^ Significantly different from C (*p* < 0.05); ^e^ Significantly different from P6.5 (*p* < 0.05); ^f^ Significantly different from P2.1 (*p* < 0.05); ^g^ Trend for significant difference from P2.1 (*p* < 0.05).

### 3.3. Relations between Insulin GLP-1 and Energy Intake with Plasma AA Concentrations

**Figure 3:**
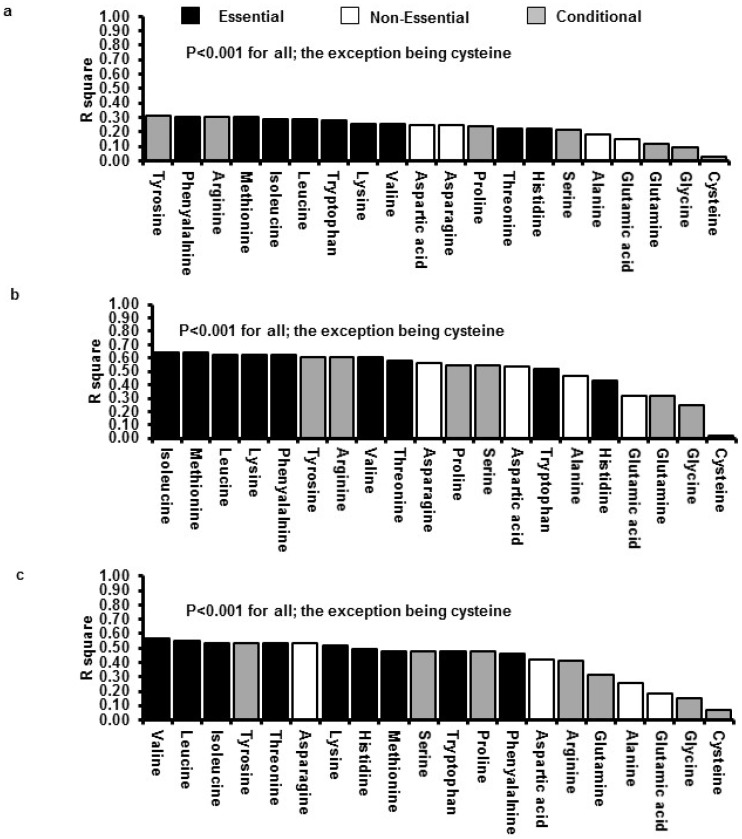
Relationships (ranked in order of strongest to weakest using *R*^2^ values) between the AUC for each of the 20 amino acids with the AUC for (**a**) energy intake; (**b**) insulin; and (**c**) glucagon-like peptide-1 (GLP-1) following the 12.5 kJ/min (P12.5) infusion of whey protein, administered at 4 mL/min for 60 min. Data are correlation coefficients for *n* = 16 subjects. Statistical significance was accepted at *p* < 0.05.

AUCs_0–60min_ of plasma concentrations for 19/20 AAs (the exception being cysteine) were related inversely to energy intake (*R*^2^ ranged between 0.09 and 0.3, *p* < 0.05), and directly to AUCs_0–60min_ of the serum/plasma concentrations of insulin (*R*^2^ ranged between 0.3 and 0.6, *p* < 0.01) and GLP-1 (*R*^2^ ranged between 0.2 and 0.6, *p* < 0.05), respectively. Figure 3 illustrates the strength of the relationships between the AUCs of each of the 20 AA profiles with energy intake as well as the AUCs of the insulin and GLP-1 profiles. The essential AAs, leucine, valine, isoleucine, lysine, methionine, and the conditionally-essential AA, tyrosine, yielded the strongest correlations with all three outcomes.

## 4. Discussion

This study has established that (i) increasing loads of ID whey protein hydrolysate led to load-dependent increases in the plasma concentrations of 19/20 free AAs over a 60-min period; (ii) plasma concentrations of the essential AAs reflected closely their concentrations in the infused whey protein, whereas the relationships were more variable for the non-essential and conditional AAs; and (iii) changes in plasma concentrations of the essential AAs, leucine, valine, isoleucine, methionine and lysine, and the conditionally-essential AA, tyrosine, were most closely associated with changes in energy intake, serum insulin and plasma GLP-1.

The first aim of this study was to determine the plasma concentrations of the 20 free AAs reaching the peripheral circulation in response to increasing loads of ID 18.1% hydrolysed whey protein. The findings in this study indicate that whey protein delivered to the duodenum at rates lower than (2.1 kJ/min), comparable to (6.3 kJ/min), and towards the upper end (12.5 kJ/min) of the normal range for gastric emptying, increased, in a load-dependent manner, the plasma concentrations of 19/20 AAs. While plasma concentrations of the majority of AAs were increased by a whey protein load as low as 2.1 kJ/min, a load of ≥6.3 kJ/min was required to increase alanine, glycine and tryptophan above control concentrations. Notably, cysteine was the only AA that was not increased by any load, confirming previous reports from studies using pig models indicating that cysteine is readily catabolised in the GI tract [22].

A limited number of studies have reported that concentrations of specific AAs reaching the peripheral circulation closely resemble their concentrations in the ingested protein source [23,24]. In contrast, other studies have found no relationships with any AA [25,26,27], or strong relationships only for the branched-chained AAs [13,14,28,29]. These discrepant observations may be accounted for by the use of high-protein drinks that included additional macronutrients, differing protein sources (*i.e.*, dairy *vs.* plant protein, and also un-hydrolysed *vs.* hydrolysed proteins), oral administration (affecting gastric emptying), differences in the load of protein administered between individuals (e.g., prescription based on a g per kg total body weight or, per kg of fat free mass), and lack of standardisation of the meal immediately preceding the study. While our findings establish strong within-subject relationships between plasma concentrations of all essential AAs with their concentration in the infused protein load, there were also strong relationships with the conditional AAs, arginine, tyrosine and proline, while relationships for the non-essential AAs, aspartic acid, alanine and glutamic acid, or the conditional AAs, serine, glutamine, and glycine, were weak to moderate. Taken together, our observations highlight existing, although poorly appreciated, knowledge that plasma AA concentrations are modulated simultaneously by many factors including (i) the rates at which “free” AAs and di- and tri-peptides are taken up by the intestinal mucosa; (ii) the digestion and absorption of endogenous small intestinal protein; (iii) metabolic transformation during absorption (which is particularly extensive for the dicarboxylic AAs, glutamic and aspartic acids); and (iv) rates of uptake and release of AAs by the liver and other tissues [30].

A further aim of this study was to determine the relationships between plasma concentrations of the 20 free AAs with the changes in serum insulin and plasma glucagon-like peptide-1 concentrations as well as energy intake in response to ID whey protein hydrolysate, reported previously [7]. Several studies have found modest associations between increased postprandial plasma concentrations for the essential AAs with either an increased release of some gut hormone (particularly GLP-1 and GIP) and/or insulin, and/or with a reduced energy intake [12,13,14]. A major strength of the current study was that the relationships of all 20 AAs with all three outcomes were assessed concurrently. This analysis was performed based on emerging evidence, in both rodents and humans, that leucine, glutamate, tryptophan, tyrosine and histidine (glutamate is a neurotransmitter and the latter three are all precursors of neurotransmitters), as well as lysine, threonine, phenylalanine, arginine and glutamine, can directly stimulate GLP-1 and insulin, and/or indirectly activate vagal pathways connecting the gut with the brain [31,32,33]. We have demonstrated that postprandial concentrations of all AAs, apart from cysteine, were correlated moderately with protein-induced suppression of energy intake, and strongly with postprandial concentrations of insulin and GLP-1. In addition, after ranking each AA based on the strength of their relationship with energy intake and AUCs of insulin and GLP-1 profiles, respectively, we observed that the essential AAs, leucine, valine, isoleucine, methionine and lysine, and the conditionally-essential AA, tyrosine, were the AAs that were most strongly correlated to all of these outcomes. While we recognise that relationships do not imply causality, our findings strongly indicate the need for further investigation of the role of these select AAs in the regulation of glucose homeostasis and energy intake using a study design that can directly determine the effects of isolated AAs.

Several aspects of our study design should be recognised when interpreting our results. Only healthy, lean males were included, and hence, the findings should not be generalised to females and obese people because they tend to be less sensitive to dietary manipulation [34], and insulin and GLP-1 concentrations and energy intake are affected by the menstrual cycle [35]. Although the total loads of protein delivered (*i.e.*, 8, 24 and 48 g, respectively) are representative of loads consumed in a snack or within a main meal, the observed findings may be different for other dairy or non-dairy protein sources, or if the protein was consumed orally or infused intragastrically. In addition, the effects of hydrolysed whey protein isolate are not necessarily generalizable to whole dairy and non-dairy protein foods which contain varying amounts of fat and carbohydrate and other dairy, or non-dairy, bioactives.

## 5. Conclusions

In conclusion, this study provides new insights into the concentrations of 20 amino acids reaching the peripheral circulation following defined loads of whey protein that were infused intraduodenally to reflect the lower, middle and upper rate of the normal gastric emptying range for nutrients in human subjects. In addition, the study has identified relationships between the concentrations of some of these amino acids (particularly leucine, valine, isoleucine, lysine, methionine and tyrosine) with specific GI and gluco-regulatory functions, as well as energy intake. Collectively, these findings make an important contribution to a comprehensive understanding of the glycaemic and satiety effects of dietary protein for the management of obesity.

## Abbreviations

ID: intraduodenalGI: Gastrointestinal
GLP-1: glucagon-like peptide-1
CCK: cholecystokinin
GIP: glucose-dependent insulinotropic polypeptide
PYY: peptide YY
AA: area amino acids
AUC_0–60 min_: area under the curve from 0 to 60 min
P2.1: protein load 2.1 kJ/min
P6.3: protein load 6.3 kJ/min
P12.5: protein load 12.5 kJ/min
C: saline control
APD: antropyloroduodenal motility
VAS: visual analog scale questionnaire
